# Student Opinions on Interdisciplinary Team Teaching of Anatomy in a Medical, Dental, and Physical Therapy School

**DOI:** 10.1002/ca.70018

**Published:** 2025-08-03

**Authors:** Jeremy J. Grachan, Thomas A. Koc, Justin Behnke, Abhishek Yadav, Sophia Chen, Christin Traba, George Holan

**Affiliations:** ^1^ Office of Education Rutgers New Jersey Medical School Newark New Jersey USA; ^2^ Department of Medicine Rutgers New Jersey Medical School Newark New Jersey USA; ^3^ Department of Physical Therapy Kean University New Jersey USA; ^4^ Department of Pediatrics Rutgers New Jersey Medical School Newark New Jersey USA; ^5^ Department of Surgery Rutgers New Jersey Medical School Newark New Jersey USA

**Keywords:** anatomy education, health professions education, interdisciplinary teaching team, small group learning

## Abstract

While some health science professional programs may utilize a team to teach anatomy, they are often anatomists or professionals from the same field. The purpose of this study was to investigate the perspectives of health professional students on the incorporation of interdisciplinary team teaching comprised primarily of an anatomist, physical therapist, and a medical doctor to collectively teach a small group anatomy curriculum. The medical students (MS; *N*_Class of 2025_ = 179 and *N*_Class of 2026_ = 174), dental students (DS; *N*_Class of 2027_ = 90), and physical therapy students (PT; *N*_Class of 2027_ = 25) were enrolled in a human anatomy course between January 2023 and December 2023 and were invited to participate in this survey. Most students affirmed that each team member provided diverse perspectives to apply the anatomy to clinical settings, offering valuable insights and guidance for upper‐level courses and their careers. They acknowledged that different team members excelled in specific content areas, and even though students did not often specifically target a faculty member for various topics, collectively, the diverse teaching team aided in facilitating a well‐rounded learning experience. Students expressed high satisfaction with their professional school anatomy course, which was co‐taught by an interdisciplinary team. Although students had access to faculty from different professions, they generally did not seek out specific faculty for field‐related inquiries. They preferred to interact with the main teaching faculty, which included the primary anatomist, medical doctor, and physical therapist.

## 1 Background

In the domain of health professions education, the study of human anatomy holds a pivotal position, offering the fundamental knowledge essential for comprehending the complexities of the human body. Traditional methods of anatomy education have served as the backbone for aspiring healthcare professionals, offering a comprehensive exploration of anatomical structures through dissections of human anatomical donors (i.e., cadavers) and didactic learning (Estai and Bunt 2016). Currently, a rising issue is the new scarcity of doctors (Ph.D.) of anatomy (Wilson et al. 2020). Since 1969, Ph. D.searned in anatomy have been steadily declining, leading to a large gap in content‐specific qualified faculty that have the capability to instruct graduate anatomy courses (Wilson et al. 2019; Wilson et al. 2020). As enrollment in health professions education continues to surge, the demand for skilled anatomists escalates proportionately. One strategy to address the demand for anatomists is to enlist individuals with related degrees, such as clinicians, to assist in teaching human anatomy or serve as educators themselves. Incorporating clinical faculty into anatomy education fosters an interdisciplinary teaching team that bridges the gap between theoretical knowledge and clinical practice (Reidenberg and Laitman 2002). Collectively, a diverse anatomy teaching team can offer expertise in both the foundational science aspects and the clinical applications, effectively preparing health professionals for their careers.

Currently, the doctoral programs at Rutgers New Jersey Medical School (MS) and Rutgers School of Dental Medicine (DS), as well as Kean University's Doctor of Physical Therapy program (PT), all utilize the same interdisciplinary team teaching approach to enhance anatomy education. Interdisciplinary can be defined as when two or more disciplines, in this case, doctors of anatomy, medical doctors, and physical therapists, pool their resources to instruct in joint courses and cooperative projects. (Parse 2014; Mahler et al. 2014). At MS, DS, and PT, anatomy is taught in a small group format that allows for all members of the teaching team to be available for students to ask questions and discuss content of clinical relevance. While these faculty are from different professions, this interdisciplinary approach differs from interprofessional education as interprofessional would allow each educator to provide a unique contribution to teaching from their own professional perspective and not necessarily work in tandem (Parse 2014; Mahler et al. 2014). As this study explores the interdisciplinary approach, numerous advantages emerge when multiple disciplines collaborate to deliver instruction that enhances and supplements one another.

Previous research has examined the concepts of “teams” and “interdisciplinary education” from both student and faculty perspectives. Team‐based teaching, or TBL, is a form of active learning that provides students the opportunity to practice course concepts in carefully formed and managed teams to solve problems (Burgess et al. 2020). For faculty, co‐teaching, if used in TBL, can provide opportunities for basic sciences and clinical disciplines to interact and learn from each other, which can also help the students integrate content (Burgess et al. 2020). This curricular approach also provides students with multiple explanations for complex cases from others in their teams and from the different faculty. Interdisciplinary teaching has been explored in anatomy before, where faculty from different disciplines collectively taught a first‐year anatomy course for students in occupational therapy, medical radiation, and human movement degree programs (Mackintosh et al. 2007). One institution had each faculty member developing and teaching sections of the course that they had a passion for or were their area of expertise and utilized students' course evaluations to gain students' opinions rather than a targeted survey (Mackintosh et al. 2007). The evaluations revealed that faculty experienced heightened feelings of collegiality, collaboration, and support, while also offering avenues for exchanging ideas, embracing diverse perspectives, and observing various teaching methods. Our study differs from Mackintosh et al. (2007) by having each member of the teaching team collaboratively work on preparing all of the course content instead of members working on specific topics. Additionally, our anatomy content is taught in a small group format where all members of the teaching team were present to assist students in their discussions. The combined approach of co‐teaching, adaptable to small group settings, along with interdisciplinary teaching, proved advantageous for both students and faculty members. The purpose of this study was to investigate student perspective on the implementation of an interdisciplinary team teaching anatomy within a small group setting, to different health professional students, including those enrolled at Rutgers New Jersey Medical School, Rutgers School of Dental Medicine, and Kean University's Doctor of Physical Therapy program. This study evaluated the students' views on learning anatomy from an interdisciplinary teaching team, gauged the students' perception of learning from each type of educator in the interdisciplinary teaching team, and explored if students choose to utilize certain educators more for different portions of the curriculum. It was hypothesized that students would be in favor of an interdisciplinary teaching team for anatomy and find the variety of backgrounds of the educators useful for specific components of the curriculum.

## 2 Methods

All methods in this study were approved by the Institutional Review Board at Rutgers University (Pro2023001669).

### 2.1 Educational Context

There are three primary members of the anatomy teaching team who are full‐time anatomy educators and who teach in all three curricula. Each member possesses a terminal degree in their respective field: a classically trained anatomist with 3 years of teaching experience, a physical therapist with 10 years of teaching experience, and a medical doctor with 26 years of teaching experience. Additionally, the teaching team includes one or two post‐doctoral fellows who are taking a research/teaching year between completing their Doctor of Medicine degree and beginning their residency. Collectively, these four to five individuals (i.e., depending on if there are 1 or 2 post‐doctoral fellows) form the main interdisciplinary teaching team. While each program is taught separately, it is taught by the same 4–5 members of the main interdisciplinary teaching team. In the medical course, the class (*n* = 180) is split into two sessions of 90 for both class discussions and dissections. There are additional faculty and residents who provide assistance during certain dissection labs focusing on content relevant to their expertise (e.g., an orthopedic surgeon aided on the days the lower limb anatomy was taught). The number of faculty and residents varies for each session but usually includes an additional 3–5 faculty per session of 90 students (i.e., for the medical curriculum, there is a total of 7–10 instructors in the dissection lab portion). For the physical therapy course, there are two additional faculty from the PT program, consisting of a physical therapist and a neuroscientist, along with two teaching assistants (3rd‐year PT students). These four individuals typically lead two or three class discussion sessions each week. The main interdisciplinary teaching team and one of the additional faculty from the PT program lead the other two or three class discussion sessions, as well as the dissection session each week. On average, there are four to five faculty at each session for the class of 25. Specific questions were added to the survey for these programs asking about these additional members of the teaching team. For the dental program, there is only the main interdisciplinary teaching team (i.e., 4–5 faculty) teaching the class of 90.

All three courses were taught at Rutgers New Jersey Medical School. Each course utilized the same teaching approach, which involved small group discussions followed by a traditional small group lab dissection using human anatomical donors. Students remain in the same groups (*n* = 6 students per group) for the duration of the course. During the small group discussions, students review an internal anatomy guide of key learning topics and discuss embedded open‐ended clinical correlation questions, known as “Questions for Application”. These questions are designed to promote active thinking of the material presented and discussions within their small groups before seeking help from a faculty member. Also, similar to team‐based learning, the students complete individual and team summative assessments before completing the respective dissection. These groups meet in a large room so they can engage with the entire team of anatomy educators during their discussions and to help answer their questions.

The medical students are taught the anatomy curriculum as part of an integrated, organ systems‐based curriculum during the first 2 years of their undergraduate medical education. In total, the medical students attend 29 formal teaching sessions during this time, 15 during the first year and 14 during the second year. The dental students are taught the anatomy curriculum in their “Gross Anatomy and Embryology” course during the first semester of their first year of dental school (i.e., 14 weeks). In total, the dental students attend 16 formal teaching sessions and 5 mandatory review sessions during this time. The physical therapy students are taught the anatomy curriculum in their “Gross and Surface Anatomy” course during the first semester of their first year of physical therapy school (i.e., 10 weeks). In total, the physical therapy students attend 17 formal teaching sessions and 4 mandatory review sessions with the primary members of the anatomy teaching team. Additionally, the physical therapy students attend 13 review sessions with just the faculty members from Kean University and two teaching assistants (3rd‐year PT students).

### 2.2 Participants

Students who were enrolled in a human anatomy course between January 2023 and December 2023 were asked to complete an informed consent and a survey that evaluated students' opinions of an interdisciplinary faculty teaching team for their human anatomy content. Medical, dental, and physical therapy students were all taught during this timeframe by the same core teaching faculty. For the medical students, this included the Class of 2025 during their second year of medical school (*n* = 179) and the Class of 2026 (*n* = 174) during their first year of medical school. The dental students (*n* = 90) were part of the Class of 2027, and the physical therapy students (*n* = 25) were part of the Class of 2026. All students enrolled in these courses were included in the study, and participation was voluntary. Any subject who completed the entire survey was included in the study.

### 2.3 Data Collection

This study utilized a primarily quantitative survey with questions that were modified from the standard template used for faculty course evaluations (i.e., Dr. _____ helped provide high quality session(s) that helped me to improve my understanding of the material), as well as other Likert‐scale and multiple choice questions developed by the research team. The survey underwent a Delphi process for content and face validity. It was reviewed by 2 medical students and 2 doctor of physical therapy students, all four of whom were previous students in an anatomy course taught by the same faculty in the same format. Any suggestions or points of clarification from these students were reviewed by the research team, and the survey was adjusted accordingly.

The anonymized survey created in Microsoft Forms was sent to students via email, with one reminder email sent 1 week after the initial email. MS Class of 2025 completed the survey at the end of their second year of medical school, MS Class of 2026 completed the survey at the end of the first year of medical school, and the DS Class of 2027 and PT Class of 2027 completed the survey at the end of their anatomy course. As part of the survey, the students were provided with a list of the common faculty members, their role, and their degree. The survey primarily used closed‐ended, 5‐point Likert scale (i.e., 1 = Strongly Disagree, 2 = Disagree, 3 = Neither Agree or Disagree, 4 = Agree, 5 = Strongly Agree) questions and open‐ended questions for feedback on the interdisciplinary teaching team. The closed‐ended questions included questions about the level of agreement regarding statements about the interdisciplinary teaching team (*n* = 10), as well as questions where they could select which member or members of the teaching team (i.e., they could select as many faculty as they would like) they preferred to ask questions to about different aspects of the anatomical content (e.g., gross anatomy, clinical topics; *n* = 4). Detailed examples of the closed‐ended questions can be found in the tables and figures within the Results section. Open‐ended questions (*n* = 5) asked for the students to leave any comments or thoughts about the utilization of each specialty of faculty member (e.g., PhD, DPT, post‐doctoral fellows).

### 2.4 Data Analysis

All numeric data was analyzed using IBM SPSS Version 29. The number of subjects and respective statistical analyses are reported for each question analyzed, including means (*M*) and standard deviations (SD). The data was screened for normality using the Shapiro–Wilk test (i.e., the data is not normally distributed if *p* < 0.05) and homogeneity of variance using Levene's test (i.e., variances are significantly different if *p* < 0.05) prior to analyses. As the data was found to be not normally distributed, a Kruskal‐Wallis *H* test was used to compare responses between programs and a Dunn's post hoc test was used to determine which groups were significantly different. Adjusted *p* values are presented for post hoc analyses. For each analysis, the data were considered statistically significant when *p* < 0.05, unless otherwise specified. To thematically analyze the open‐ended questions, all the responses were reviewed by two co‐authors to decide on common qualitative themes. Each author followed the first four steps outlined in Braun and Clare's (2006) six‐step approach to thematic analysis individually. At the end of the individual analyses, the two authors compared the themes they developed and any discrepancies in themes were discussed and solved by consensus of the two reviewers to complete the last two steps of the analysis.

## 3 Results

**FIGURE 1:**
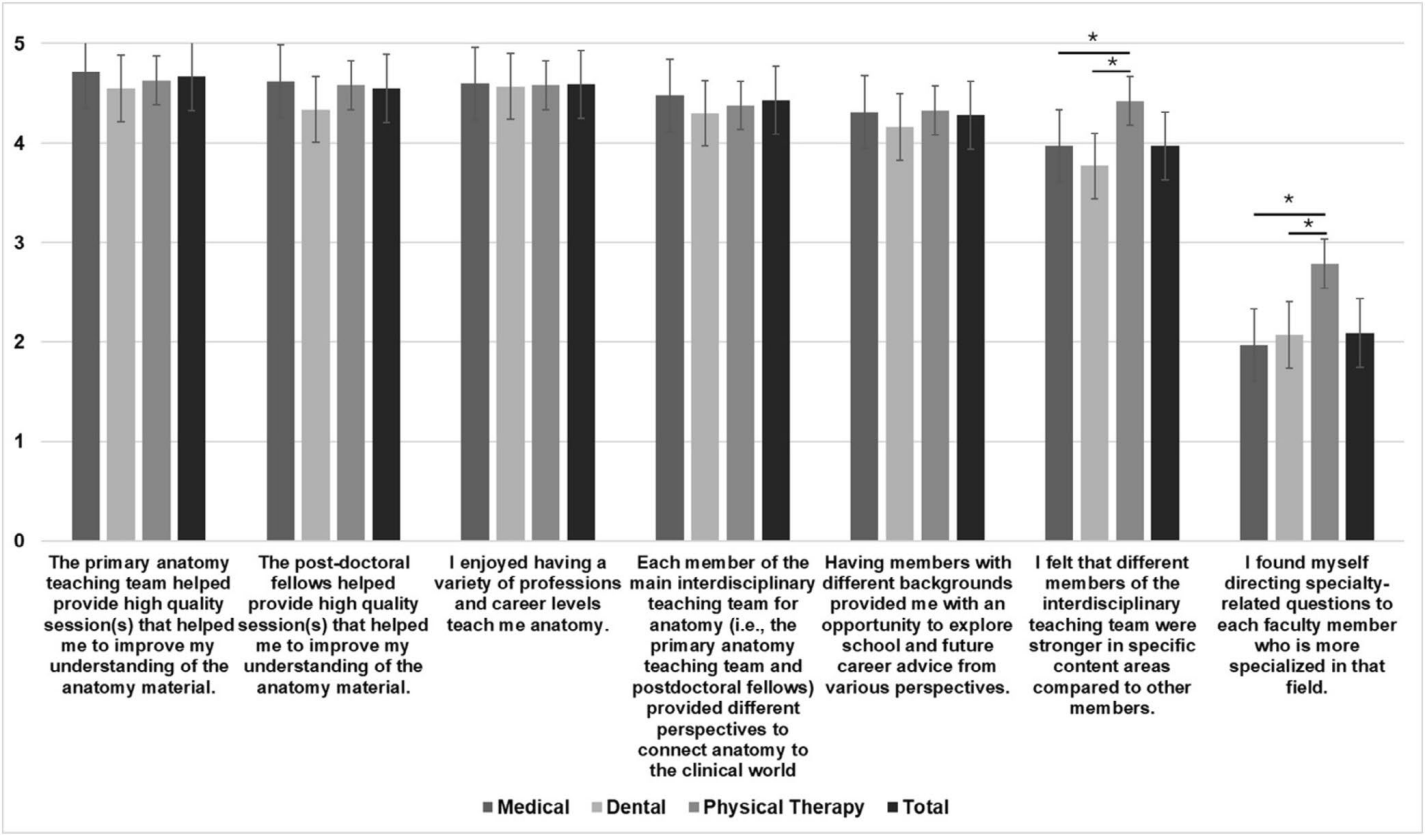
Combined (MS, DS, and PT) student opinions of the main anatomy interdisciplinary teaching team (*n* = 239). Each statement was rated on a 5‐point scale (1 = *Strongly Disagree*, 5 = *Strongly Agree*) and was significant if *p* < 0.05(*).

Please note that the primary teaching team includes the main anatomist, main physical therapist, and the main medical doctor, whereas the main interdisciplinary anatomy teaching team includes the primary teaching team and post‐doctoral fellows. The results are organized by respective programs, with the tables and figures presenting the programs combined. Figure 1 summarizes the students responses related to the interdisciplinary team, Table 1 summarizes students preferred faculty member to ask questions related to specific topics (which students could select as many answers as they wanted), and Table 2 is a summary of the themes of the open‐ended responses with examples from the different programs.

**TABLE 1:** Number of students who preferred faculty members for different question topics (*n* = 239)^a^.

Survey question	MS (*n* = 159)	DS (*n* = 56)	PT (*n* = 24)	Total (*n* = 239)
Gross anatomy content				
Main physical therapist	151 (95.0%)	46 (82.1%)	24 (100.0%)	221 (92.5%)
Main anatomist	138 (86.8%)	45 (80.4%)	14 (58.3%)	197 (82.4%)
Main medical doctor	93 (58.5%)	49 (87.5%)	24 (100.0%)	166 (69.5%)
Main post‐doctoral fellow(s)	53 (33.3%)	26 (46.4%)	13 (54.2%)	92 (38.5%)
Clinical faculty/residents	28 (17.6%)	—	—	—
Additional physical therapist	—	—	9 (37.5%)	—
Additional physical therapist and neuroscientist	—	—	2 (8.3%)	—
Teaching assistants	—	—	8 (33.3%)	—
Anatomical dissections				
Main physical therapist	143 (89.9%)	39 (69.6%)	23 (95.8%)	205 (85.8%)
Main anatomist	129 (81.1%)	38 (67.9%)	21 (87.5%)	188 (78.7%)
Main medical doctor	116 (73.0%)	49 (87.5%)	22 (91.7%)	187 (78.2%)
Main post‐doctoral fellow(s)	42 (26.4%)	24 (42.9%)	5 (20.8%)	71 (29.7%)
Clinical faculty/residents	31 (19.5%)	—	—	—
Additional physical therapist	—	—	0 (0.0%)	—
Additional physical therapist and neuroscientist	—	—	0 (0.0%)	—
Teaching assistants	—	—	0 (0.0%)	—
Clinical topics (i.e., condition, treatments, surgical procedures)				
Main physical therapist	108 (67.9%)	43 (76.8%)	22 (91.7%)	173 (72.4%)
Main anatomist	88 (55.3%)	33 (58.9%)	12 (50.0%)	133 (55.6%)
Main medical doctor	76 (47.8%)	40 (71.4%)	11 (45.8%)	127 (53.1%)
Main post‐doctoral fellow(s)	68 (42.8%)	23 (41.1%)	8 (33.3%)	99 (41.4%)
Clinical faculty/residents	81 (50.9%)	—	—	—
Additional physical therapist	—	—	15 (62.5%)	—
Additional physical therapist and neuroscientist	—	—	2 (8.3%)	—
Teaching assistants	—	—	6 (25.0%)	—
Connecting anatomy to how it would be used in their future (e.g., boards, treating patients, interdisciplinary teamwork)				
Main physical therapist	103 (64.8%)	42 (75.0%)	22 (91.7%)	167 (69.9%)
Main anatomist	82 (51.6%)	33 (58.9%)	7 (29.2%)	122 (51.0%)
Main medical doctor	67 (42.1%)	31 (55.4%)	9 (37.5%)	107 (44.8%)
Main post‐doctoral fellow(s)	72 (45.3%)	23 (41.1%)	7 (29.2%)	102 (42.7%)
Clinical faculty/residents	86 (54.1%)	—	—	—
Additional physical therapist	—	—	20 (83.3%)	—
Additional physical therapist and neuroscientist	—	—	5 (20.8%)	—
Teaching assistants	—	—	7 (29.2%)	—

Abbreviations: DS, dental school; MS, medical school; PT, doctor of physical therapy program.

^a^ Students could select as many answers as they would like for each question.

**TABLE 2:** Themes from open‐ended responses about the interdisciplinary teaching team.

Theme	Sample quotations
Diversity in professional backgrounds among faculty provides opportunities for different learners to relate to the subject matter	“It's crucial to have different perspectives from different fields for a better understanding.”—*MS Student* “I really appreciate having professionals from different backgrounds teaching anatomy because they all bring such different perspectives to the same topics and it helps me understand them more.”—*DS Student* “Different professors have different experiences so it was nice to hear it from all of them.”—*PT Student*
Diversity in professional backgrounds among faculty provides opportunities for learners to benefit from specific expert knowledge	“Appreciated that [medical doctors] could give clinical background for relevant topics, such as the anatomy involved in procedures, surgeries, etc.”—*MS Student* “I appreciate having medical doctors because they have studied the body and are able to apply clinical situations to help enforce the topics.”—*DS Student* “I believe having medical doctors in the class was very helpful as their wider range of knowledge offered different perspectives and explanations that were helpful to approaching topics I found confusing.”—*PT Student* “I really appreciated having a physical therapist because was extremely helpful when it came to understand the muscles and their innervations as well as the other topics especially in the dissection portion”—*DS Student* “I think during the dissections, I had a lot of help from the anatomy educators and I could better distinguish between structures and learn about anatomical variations in the donors.”—*PT Student* “[The anatomist was] able to provide solid ideas, concepts and learning strategies towards understanding information more than clinicians who often just knew the information without knowing how to teach it.”—*MS Student*
Inclusion of near‐peer educators provides opportunities for early professional learners to gain level‐appropriate insight and mentorship	“It was great having more consistent relationships with medical fellows who can give advice on medical school/residency apps. As an M1 I really appreciated this.”—*MS Student* “Extremely helpful because this is their thing and they talk about what their journey has been like and more surgical/procedural parts of medicine and anatomy.”—*MS Student* “Make you feel very comfortable in asking questions and have more ‘modern’ way of memorizing certain things.”—*PT Student* “The 3rd year TA's were very approachable and helped us with any which way they could both inside and outside of class.”—*PT Student*

Abbreviations: DS, dental school; MS, medical school; PT, doctor of physical therapy program.

### 3.1 Medical Student (MS) Responses

#### 3.1.1 Response Rate

The MS Class of 2025 had 179 students enrolled, with 62 (34.6%) who responded to the survey. Similarly, the MS Class of 2026 had 174 students enrolled, with 97 (55.7%) responding to the survey.

#### 3.1.2 Medical Student Opinions of the Anatomy Interdisciplinary Teaching Team

Most medical students agreed or strongly agreed that the primary teaching team helped provide high quality sessions that improved their understanding of the anatomy material (*M* = 4.72; SD = 0.70). Similarly, they enjoyed having a variety of professions and career levels teaching them anatomy (*M* = 4.60; SD = 0.61) and felt that each member provided different perspectives to connect anatomy to the clinical world (*M* = 4.48; SD = 0.64). In addition to the main interdisciplinary teaching team, medical students were also taught anatomy with the help of other clinical faculty/residents from various specialties (e.g., Surgery, Orthopedics, PM&R). Specifically, these additional individuals would come and help during the dissection components of the anatomy sessions. Students agreed that the inclusion of these additional members improved their understanding of the anatomy material (*M* = 4.17, SD = 0.81).

#### 3.1.3 Medical Students' Faculty Preferences for Parts of the Anatomy Curriculum

In addition to getting the students' perceptions and opinions of the interdisciplinary teaching team, students were provided with four topics related to the anatomy course and asked to select each faculty member they preferred to ask questions related to these topics to, with the ability to select each faculty member that applied. For gross anatomy content, medical students (*n* = 159) preferred to ask the main physical therapist (95.0%) or the main anatomist (86.8%). When asking questions related to the anatomical dissections, the medical students again preferred to ask the main physical therapist (89.9%) or the main anatomist (81.1%). For questions related to clinical topics, medical students tended to ask the main physical therapist (67.9%) or the main anatomist (55.3%), but were also inclined to ask the clinical faculty/residents (50.9%), main medical doctor (47.8%), or the post‐doctoral fellow(s) (42.8%). Finally, in regard to who they preferred to ask questions relating anatomy and how it would be used in their future, most medical students chose to ask the main physical therapist (64.8%), the clinical faculty/residents (54.1%), or the main anatomist (51.6%).

### 3.2 Dental Student (DS) Responses

#### 3.2.1 Response Rate

For the DS Class of 2027, 56 (62.2%) responded to the survey out of the 90 enrolled.

#### 3.2.2 Dental Student Opinions of the Anatomy Interdisciplinary Teaching Team

Similar to medical students, most dental students agreed or strongly agreed that the primary teaching team helped provide high quality sessions that improved their understanding of the anatomy material (*M* = 4.55; SD = 0.78), enjoyed having a variety of professions and career levels teach them anatomy (*M* = 4.57; SD = 0.57), and felt that each member provided different perspectives to connect anatomy to the clinical world (*M* = 4.30; SD = 0.81).

#### 3.2.3 Dental Students' Faculty Preferences for Parts of the Anatomy Curriculum

Dental students (*n* = 56) preferred to ask either the main medical doctor (87.5%), the main physical therapist (82.1%), or the main anatomist (80.4%) about gross anatomy content. For anatomical dissections questions, most dental students preferred to ask the main medical doctor (87.5%), whereas for clinical topics, they preferred to ask the main physical therapist (76.8%) or the main medical doctor (71.4%). For questions relating to anatomy and how it would be used in their future, most dental students chose to ask the main physical therapist (75.0%).

### 3.3 Physical Therapy Student (PT) Responses

#### 3.3.1 Response Rate

For the PT Class of 2027, 25 students were enrolled, and 24 responded (96%) to the survey.

*Physical Therapy Student Opinions of the Anatomy Interdisciplinary Teaching Team*.

Most physical therapy students agreed or strongly agreed that the primary teaching team helped provide high quality sessions that improved their understanding of the anatomy material (*M* = 4.63; SD = 0.88). They felt that each member provided different perspectives to connect anatomy to the clinical world (*M* = 4.38; SD = 0.88). The physical therapy students also noted that they felt different members of the team were stronger in specific content areas compared to others (*M* = 4.42, SD = 0.97) and they sometimes found themselves directing specialty‐related questions to faculty members who specialized in that field (*M* = 2.79, SD = 0.93).

#### 3.3.2 Physical Therapy Students' Faculty Preferences for Parts of the Anatomy Curriculum

For gross anatomy content, physical therapy students (*n* = 24) preferred to ask questions to the main physical therapist (100.0%) or the main medical doctor (100.0%). Similarly, when asking questions related to the anatomical dissections, the physical therapy students preferred to ask the main physical therapist (95.8%) or the main medical doctor (91.7%), as well as the main anatomist (87.5%). For this topic, they noted they did not prefer to ask the additional physical therapists or teaching assistants (0.0%). For questions related to clinical topics, physical therapy students overwhelmingly tended to ask the main physical therapist (91.7%). Lastly, in regard to whom they preferred to ask questions relating to anatomy and how it would be used in their future, most physical therapy students chose to ask the main physical therapist (91.7%) or the additional physical therapist (83.3%).

### 3.4 Summary of Combined Responses

#### 3.4.1 Student Opinions of the Anatomy Interdisciplinary Teaching Team

In total, 239 students responded to the survey from across the three programs. Across all the programs, most students strongly agreed that the primary anatomy teaching team (i.e., the three full‐time anatomy faculty; *M* = 4.67, SD = 0.74) and post‐doctoral fellows (*M* = 4.43, SD = 0.86) helped improve their understanding of the anatomy material and that the students enjoyed having a variety of professions and career levels teach them anatomy (*M* = 4.59, SD = 0.63). Most students highly agreed that each member provided different perspectives to connect anatomy into clinical settings (*M* = 4.43, SD = 0.71) and that having members with different backgrounds provided students an opportunity to explore school and future career advice from various perspectives (*M* = 4.28, SD = 0.82). Lastly, students agreed that different members of the team were stronger in specific content areas compared to other members (*M* = 3.97, SD = 0.87) but that they did not find themselves directing specialty‐related questions to each faculty member who was more specialized in that field (*M* = 2.09, SD = 0.94).

A Kruskal‐Wallis *H* test was conducted for each survey question to see if there was a significant difference in responses between the different curricula (Figure 1). A statistically significant difference was found for two of the questions. The first question was if students perceived that different members of the team were stronger in specific content areas compared to other members, *Χ* ^2^(2) = 11.551, *p* = 0.003. Pairwise comparison using Dunn's procedure with Bonferroni correction for multiple comparisons found that physical therapy students (median = 5) agreed significantly more than both medical students (median = 4, *z* = −43.1, *p* = 0.007) and dental students (median = 4, *z* = −51.9, *p* = 0.003). The second question was if students found themselves directing specialty‐related questions to each faculty member who is more specialized in that field. Similarly, physical therapy students (median = 3) agreed significantly more (*Χ* ^2^(2) = 16.319, *p* < 0.001) than both medical students (median = 2, *z* = −56.6, *p* = 0.000) and dental students (median = 2, *z* = −54.8, *p* = 0.002).

In addition to the Likert‐scale questions, students were also able to share any thoughts they had on having an interdisciplinary team teach them anatomy. Common themes from the responses can be found in Table 2.

#### 3.4.2 Students' Faculty Preferences for Parts of the Anatomy Curriculum

For gross anatomy content, when averaging the student selections from across the three programs, most students preferred to ask questions to the main physical therapist (92.5%) and main anatomists (82.4%). For anatomical dissections, most students preferred to ask questions to the main physical therapist (85.8%), main anatomists (78.7%), and the main medical doctor (78.2%). For questions related to clinical topics, most students preferred to ask questions to the main physical therapist (72.4%), main anatomists (55.6%), the main medical doctor (53.1%), and the main post‐doctoral fellow (41.4%). Lastly, students had varying responses when asked who they preferred to ask questions relating to anatomy and how it would be used in their future, noting the main physical therapist (69.9%) the most, but also the main anatomists (51.0%), the main medical doctor (44.8%), and the main post‐doctoral fellow (42.7%).

## 4 Discussion

This study aimed to investigate student perspectives regarding the utilization of an interdisciplinary team co‐teaching anatomy for allopathic medical, dental, and physical therapy students. The novelty of this unique teaching team, which primarily included an anatomist, physical therapist, and medical doctors, was their collective roles in curriculum development and for co‐teaching in the classroom for the small‐group discussions and laboratory sessions.

### 4.1 Medical Student Findings

Most medical students agreed or strongly agreed that the primary teaching team helped provide high‐quality sessions that improved their understanding of the anatomy material. Additional members of the teaching team, including clinical staff, contributed to students' understanding of anatomy material, but students still tend to gravitate towards the main teaching team for questions related to anatomy content. This is consistent with McDonald et al. (2020), finding that the introduction of a team‐teaching format improved physiotherapy and exercise science/physiology student outcomes, especially for the students performing at lower academic levels.

### 4.2 Dental Student Findings

Similar to medical students, most dental students agreed or strongly agreed that the primary teaching team helped provide high quality sessions that improved their understanding of the anatomy material. One dental student in our study noted, “I really appreciate having professionals from different backgrounds teaching anatomy because they all bring such different perspectives to the same topics and it helps me understand them more.” Another study focused on interprofessional education with faculty and students from both dental and dental hygiene programs and found that, overall, students had a more positive perception of interprofessional education than before (McGregor et al. 2018). Although our programs lack an interprofessional anatomy course, the involvement of an interdisciplinary teaching team may have contributed to the positive experiences across the various programs (MS, DS, PT) examined in this study. Additionally, it fostered positive perceptions towards interprofessional collaboration.

### 4.3 Physical Therapy Student Findings

Furthermore, statistical analysis revealed significant differences in responses between programs, with physical therapy students agreeing more that team members were stronger in specific content areas and that the students found themselves directing specialty‐related questions to specialized faculty members. This variation in class size directly impacts the student‐to‐faculty ratio, potentially influencing the accessibility of resources and the dynamics of educational interactions within each discipline. Particularly, the PT program, with its significantly smaller class size, boasts a more favorable student‐to‐faculty ratio, which may afford students greater ease of access to faculty support and identifying discrepancies in content comprehension. As seen in Figure 1, physical therapy students agreed significantly more than medical and dental students that they would tailor specialty questions toward faculty who were more specialized in that field of content. It should also be noted that, as seen in Table 1, physical therapy students were seen to ask many of their clinical topics and anatomy‐based questions directly to the main physical therapist. This data suggests that the PT students may have felt more comfortable and inclined to ask someone within their aspired field.

### 4.4 Combined Responses Across Programs

Most students affirmed that each team member provided diverse perspectives to apply the anatomy to clinical settings, offering valuable insights into routes for upper‐level courses and their careers. They also acknowledged that different team members excelled in specific content areas, and even though students did not often specifically target a faculty member for various topics, collectively, the diverse teaching team aided in facilitating a well‐rounded learning experience.

This study observed notable differences in class sizes, with medical cohorts consisting of 178 students, dental cohorts consisting of 90 students, and physical therapy cohorts limited to just 25 individuals. This variation in class size directly impacts the student‐to‐faculty ratio, potentially influencing the accessibility of resources and the dynamics of educational interactions within each discipline. However, all the students from different cohorts who participated in the small group learning for anatomy with the interdisciplinary teaching team viewed it as a positive addition to their education. Others exploring this topic could see differing levels of success based on the student cohort and interdisciplinary teaching team.

While interdisciplinary teaching teams have been used before for professional school education (McDonald et al. 2020), the current study presents a specific team who (1) collaborated to develop the course content rather than divide the content across each member and (2) applied this teaching approach in a version of small‐group learning where all of the small groups met together in a larger classroom for their discussions and had the opportunity to interact with a faculty member of their choosing compared to a singular, assigned small‐group facilitator. Through the use of the group interactions within the interdisciplinary instruction, students were encouraged to work together to learn and apply the material of the content area being focused on.

### 4.5 Faculty Preferences by Topic

However, when a question became too big for the group to conquer, as seen in Figure 1, all student cohorts enjoyed the opportunity to utilize the interdisciplinary team for aid in their learning experience. This can be supported by Zheng and Zhang (2020), who found that while the use of cognitive strategy rehearsal (mental repetition) showed a negative association with student learning outcomes, employing resource management strategies, more specifically peer learning and help‐seeking, was positively correlated with academic success. Responses to open‐ended questions further underscored the benefits of peer learning and help‐seeking in a flipped classroom setting (Zheng and Zhang 2020; Sterpu et al. 2024). The current study's anatomy curriculum utilized a similar concept for the “Questions for Application” to lead students to engage in active thinking of the material presented and encourage discussions within their small groups before seeking help from a faculty member.

### 4.6 Possible Benefits for Anatomist Shortage

Given the high student satisfaction reported in this study, there is a strong potential for this curriculum model to be implemented in anatomy courses across academic levels. Recent studies have begun to address the challenge of the declining presence of anatomy doctoral programs and the resulting concerns of a shortage of formally trained anatomists (Wilson et al. 2019, 2020). With this, research has explored the optimization of TBL for anatomy education with a larger student to faculty ratio. Sterpu et al. (2024) found no differences between team‐based learning and traditional lecture groups in relation to student satisfaction for fifth‐year medical students attending the obstetrics and gynecology clerkship in Sweden, despite the TBL group having double the student‐to‐faculty ratio. These results show favorable evidence to suggest there can be ways to aid in reducing faculty pressure and workload without the worry of decreased student performance. Collectively, the data presented in the current study and the data found by others show that team teaching can be an effective method for anatomy education, with benefits for both the students and faculty alike.

### 4.7 Limitations

The limitations of this study include that the data reported is specific to one institution that has the same teaching faculty for anatomy across academic programs. Also, some of the teaching team may not have been present for several sessions across a course due to other responsibilities (e.g., other teaching or attending conferences). During the current study, some faculty were absent from some of the small group sessions. While the effect of these absences and transitions should be minor, they are a limitation nonetheless as students were not able to interact with specific faculty members as much. Other limitations to note are student perceptions of individual faculty members. This study did not account for whether specific students preferred certain faculty members over one another. Reasons why this could be a limitation are that the doctor of physical therapy is the Director of Anatomy Teaching and serves as the Course Director for each course, which may make more students inclined to ask him questions under the perception he has more control over the assessments. Also, certain students may have had varying experiences with different faculty, whether that be positive or negative, which may affect who they choose to interact with rather than the faculty member's specialty, training, or years of experience. Additionally, demographic information (e.g., gender identity, educational levels) was not collected for these participants and therefore could not be used in the analysis.

The study is also subjected to the risk for type I and II errors, which may limit the generalizability of the findings. The study does contain a relatively smaller sample size, as data was collected through non‐random single‐institution sampling, leading to an increased risk of type I and II error. Additionally, potential type I and II errors are typically more prevalent in self‐reported questionnaires/perceptions due to individual subjectivity (personal preferences, emotion, prior experiences), as well as various biases such as recall bias. Furthermore, due to the data being collected after the student's course concluded, biases may limit the objectivity and reproducibility of the findings.

### 4.8 Future Directions

In this study, participants included physical therapy and medical students who benefited from instruction delivered by both anatomists and seasoned professionals within their respective disciplines. Conversely, there was not a dentist during the anatomy education sessions, including for the DS anatomy course. The anatomy curriculum at DS does not focus on dental‐specific anatomy as the dental students take a separate course taught by dental faculty for this vital area for their careers. The inclusion of a dentist on the anatomy team, both for the whole DS course and for parts of the head and neck anatomy of the PT and MS curricula, would provide unique opportunities to correlate anatomical knowledge to the field of dental medicine and provide natural opportunities for interdisciplinary teaching activities. To address this deficiency, future efforts could focus on supplementing anatomy education with faculty who actively practice in the relevant disciplines or explore the formal and regular integration of faculty possessing clinical experience into the anatomy classroom. Additionally, this study focused solely on the students' perceptions of the interdisciplinary team. Future studies could explore more about the group dynamics during the small group discussions and the relationships to this and the interdisciplinary teaching team, as well as the effect this type of teaching has on academic performance.

## 5 Conclusion

In conclusion, our study found that students were highly satisfied with their professional school anatomy course being co‐taught by an interdisciplinary team. Despite the presence of various professions, students typically did not target specific faculty for field‐related questions. Although students agreed there were benefits to having various professions and career levels teach them anatomy, the preference for engaging with the main faculty underscores their pivotal role in shaping the students' educational experiences. However, the overall student experience was enhanced by the diverse expertise within the interdisciplinary team who offered valuable and multifaceted perspectives on the anatomy content.

## Ethics Statement

The materials and methodology for this study were approved by the Institutional Review Board (IRB) at Rutgers Biomedical and Health Sciences (Pro2023001669).

## Data Availability

The data that support the findings of this study are available from the corresponding author upon reasonable request.
